# Age of Acquisition Effects on Word Processing for Chinese Native Learners’ English: ERP Evidence for the Arbitrary Mapping Hypothesis

**DOI:** 10.3389/fpsyg.2017.00818

**Published:** 2017-05-18

**Authors:** Jin Xue, Tongtong Liu, Fernando Marmolejo-Ramos, Xuna Pei

**Affiliations:** ^1^School of Foreign Studies, University of Science and Technology BeijingBeijing, China; ^2^School of English Language, Literature and Culture, Beijing International Studies UniversityBeijing, China; ^3^Department of Psychology, Stockholm UniversityStockholm, Sweden; ^4^Faculty of Health Sciences, The University of Adelaide, AdelaideSA, Australia

**Keywords:** AoA, event-related potentials, semantic processing, Chinese-native learners of English, arbitrary mapping

## Abstract

The present study aimed at distinguishing processing of early learned L2 words from late ones for Chinese natives who learn English as a foreign language. Specifically, we examined whether the age of acquisition (AoA) effect arose during the arbitrary mapping from conceptual knowledge onto linguistic units. The behavior and ERP data were collected when 28 Chinese-English bilinguals were asked to perform semantic relatedness judgment on word pairs, which represented three stages of word learning (i.e., primary school, junior and senior high schools). A 3 (AoA: early vs. intermediate vs. late) × 2 (regularity: regular vs. irregular) × 2 (semantic relatedness: related vs. unrelated) × 2 (hemisphere: left vs. right) × 3 (brain area: anterior vs. central vs. posterior) within-subjects design was adopted. Results from the analysis of N100 and N400 amplitudes showed that early learned words had an advantage in processing accuracy and speed; there is a tendency that the AoA effect was more pronounced for irregular word pairs and in the semantic related condition. More important, ERP results showed early acquired words induced larger N100 amplitudes for early AoA words in the parietal area and more negative-going N400 than late acquire words in the frontal and central regions. The results indicate the locus of the AoA effect might derive from the arbitrary mapping between word forms and semantic concepts, and early acquired words have more semantic interconnections than late acquired words.

## Introduction

Age of acquisition (AoA) refers to the age at which a concept or a skill is learned (Hernandez and Li, 2007). Early-learned words have advantage over late AoA words in processing accuracy and speed (Brysbaert et al., 2000; Zevin and Seidenberg, 2002; Belke et al., 2005; Hernandez et al., 2007; Bowers and Kennison, 2011). AoA is an important variable for lexical processing in both the first language (L1) (Brysbaert et al., 2000; Mayberry and Lock, 2003; Weekes et al., 2004; Belke et al., 2005; Chen et al., 2007) and the second language (L2) (Bowers and Kennison, 2011; DeKeyser, 2013; Llanes and Muñoz, 2013; Saito, 2013; Stolten et al., 2013; Marcotte and Ansaldo, 2014; Montrul and Foote, 2014). Relatively little empirical evidence is devoted to the L2 AoA effect for Chinese native learners who learn L2 English as a foreign language. The present study was aimed at examining the L2 AoA effect on word processing in native Chinese learners of English.

L2 AoA is observed to correlate negatively with L2 language proficiency mostly in naturalistic settings (Birdsong, 2005; Saito, 2013; Montrul and Foote, 2014). Despite the evidence of advantage of early language learning, however, a large body of research suggests L2 learning depends on several factors such as language experience (Rakhlin et al., 2015), affect (Pfenninger and Singleton, 2016), quality and quantity of language input (Flege et al., 1999). Early start does not confer the kinds of advantage one might expect. Especially, previous evidence has not confirmed the long-term benefits of an early start (Muñoz, 2008). So far, empirical evidence on the effect of early L2 learning is still rare for Chinese natives who learn English mostly via classroom instruction. To the best of our knowledge, only two empirical studies addressed the L2 AoA effect with Chinese native learners of English (Lu and Tu, 2010; Huang, 2014), and results were controversial. Lu and Tu (2010)’s study examined whether L2 AoA influenced the mental lexicon representation of high-proficiency Chinese-English bilinguals by using a Bilingual Stroop Task. Results of this study failed to find the magnitude difference in the between-language interference between the early and late bilinguals. Huang (2014)’s study administered L2 English grammar and speech production to 118 Mandarin-speaking immigrants in the United States. Results showed that the age of learning had a robust effect on both L2 grammar and speech production after controlling length of residence and years of education in the United States. The primary goal of the present study was to examine whether neural processing patterns of early learned L2 words distinguish from late learned L2 words for Chinese natives who learn English as a foreign language in China. Both behavioral and EEG data were collected to examine the L2 AoA effect in the present study.

The technique of Event-related potentials (ERPs) has an advantage in capturing at the millisecond level the neural activities that respond to different experimental manipulations (Yang et al., 2007). The different patterns of brain responses are represented by polarities, latencies, amplitudes, and topography of ERPs (Kutas and Federmeier, 2001). An examination of the electrophysiological brain responses would reflect the lexical, pre-lexical or post-lexical locus of an effect, as well as its orthographic, phonological or semantic nature (Cuetos et al., 2009). Accordingly, if a variable like AoA produces activation at a time window, it can be inferred that it corresponds to a certain language process.

Neurocognitive research has detected AoA-related neuronal changes in language processing. Learning an L2 after gaining proficiency in L1 was found to modify brain structure in an AoA dependent manner (Klein et al., 2014). To be specific, later onset of L2 learning is associated with significantly thicker cortex in the left inferior frontal gyrus (IFG) and thinner cortex in the right IFG. A recent MRI study suggests that volumetric measures of the right angular gyrus (AG) and right superior parietal lobule (SPL) plus the cortical area of right SPL in the parietal lobe were reliably sensitive to L2 AoA (Wei et al., 2015). Earlier second language exposure was associated with larger volumes in the right parietal cortex and the cortical area of the right SPL increased as AoA decreased. On the other hand, the AoA-related difference in brain substrates modulates functional neural activity in several aspects of language processing such as grammar and semantic access (Weber-Fox and Neville, 1996; Wartenburger et al., 2003). For instance, an ERP study found differences between native speakers and early L2 learners for syntactic processing (AoA between 1 and 3 years); in contrast, differences between monolinguals and L2 speakers were observed only in individuals who learned L2 after the age of 11 for semantic processing (Weber-Fox and Neville, 1996). Differential recruitment of brain regions was associated with grammatical processing especially for late L2 learners, and neuronal activity was greater for irregular grammatical items than regular items (Wartenburger et al., 2003).

Several approaches to the AoA effect have been proposed. *The phonological completeness hypothesis* proposes that early learned vs. late learned words are stored differently in the speech output lexicon, with the former in a holistic way and the latter in a fragmented way (Brown and Watson, 1987). *The Semantic Locus Hypothesis* (Brysbaert et al., 2000; Belke et al., 2005) claims that late learned words are incorporated into the semantic representation already existing in the early learned words. The speed and efficiency in semantic activation are determined by the order of acquisition. Thus, early learned concepts are more accessible than late learned concepts. However, the semantic locus and phonological completeness accounts do not predict L2 AoA effects on phonological, syntactic, and semantic processing (for detail discussions see Hernandez and Li, 2007). Another line of study revealed language processing was differential across age groups and language groups, and this was bounded in the sensorimotor system (Binder and Desai, 2011; Iossifova and Marmolejo-Ramos, 2013; Xue et al., 2014, 2015; D’Angiulli et al., 2015), thus attributing the AoA effect to the neural and computational mechanisms underlying learning and sensorimotor integration (Hernandez and Li, 2007).

Recently, *the Arbitrary Mapping Hypothesis* has been proposed to account for the locus of the AoA effect (Zevin and Seidenberg, 2002; Chen et al., 2007; You et al., 2009). This theory claims the AoA effect reflects the arbitrary nature of the mapping between input (e.g., orthography) and output (phonological or semantic) representations formed during the development of the lexical network (Belke et al., 2005; Chen et al., 2007). The AoA effect will be increased if the mapping between orthography and phonology / semantic representation (O-P/S) is arbitrary or inconsistent. The AoA effects will be reduced if the O-P/S mapping is consistent. Accordingly, the larger AoA-effect yielded from picture naming than word naming derives from the arbitrary mapping between concepts and linguistic units in picture naming; whereas, word naming involves a relatively regular mapping between orthographic and phonological representations. In the same vein, consistent O-P mappings (e.g., bake in the word family cake, flake, lake, make, stake and take) should lead to smaller AoA effects (as -ake is consistent in its spelling-sound correspondences in the orthographic family). Results from computational modelling (Ellis and Lambon Ralph, 2000) and substantial behavioral evidence suggests that the AoA effect might arise because the lexical units, also called lemmas, are arbitrarily mapped onto conceptual units (Belke et al., 2005; Chen et al., 2007). For instance, there was a larger AoA effect for Chinese characters from low-predictive families (in terms of predictability from orthography to pronunciation) than from high-predictive families in both word naming and semantic judgment tasks (Chen et al., 2007). In a study using a semantic blocking paradigm in which participants were required to name objects from the same or from different semantic categories, semantic context effects were found to be more pronounced for homogeneous sets of late-acquired words than for homogenous sets of early-acquired words (Belke et al., 2005). The interaction between AoA and naming context was interpreted as arising from the more powerful competitors of the early-acquired words during lexical-semantic encoding.

Chinese and English belong to two language systems that are distant from each other in terms of O-P mapping relationship. English word reading largely relies on the grapheme-phoneme correspondence rules (Coltheart et al., 2001; Lee et al., 2005). Comparatively, in Chinese writing system, about 81% of Chinese characters in modern Chinese consist of a semantic radical (suggesting the meaning) and a phonetic component (indicating the sound) (Li and Kang, 1993). Metalinguistic awareness of the semantic and phonetic components has a facilitative effect on Chinese character reading (Ku and Anderson, 2001). Thus, it is interesting to ask how Chinese natives take advantage of English O-P correspondence rules when they process words of different AoAs. Specifically, the second goal of the present research was to examine the Arbitrary Mapping Hypothesis (Zevin and Seidenberg, 2002) on the present sample of Chinese native leaners of English by exploring how the L2 AoA effect arose from the mapping between orthographic, and phonological/semantic representations.

To summarize, AoA seems to modulate behavior and neural response during language processing (e.g., Bowers and Kennison, 2011; Wei et al., 2015). The AoA effect has at least three possible loci: at the level of phonological access, at the semantic level, or at multiple levels, as proposed by the arbitrary mapping hypothesis (e.g., Belke et al., 2005; Chen et al., 2007; Cuetos et al., 2009). The AoA effect been examined scarcely on Chinese native learners of English. The present study examined the AoA effect on Chinese natives who learn English as a foreign language in China via classroom instruction. Two research questions were addressed: whether early vs. late learned L2 words processing elicited different behavioral and neuronal processing patterns; if yes, we then asked how O-P mapping regularity modulated the AoA effect during on-line word processing.

Previous studies in this field have usually adopted between-groups designs (e.g., Bowers and Kennison, 2011; Klein et al., 2014; Montrul and Foote, 2014), in which the AoA effect is easily confounded with some factors such as length of language study, language proficiency, maturity, etc. In the present study, the AoA and regularity was manipulated to examine how O-P mapping rules influenced the AoA effects. The same group of Chinese native learners of English were asked to make a semantic relatedness judgment on English word pairs, which represented three stages of word learning (i.e., three AoAs; for details see the materials section). A 3 (AoA: early vs. intermediate vs. late) × 2 (regularity: regular vs. irregular) × 2 (semantic relatedness: related vs. unrelated) × 2 (hemisphere: left vs. right) × 3 (brain area: anterior vs. central vs. posterior) within-subjects design was adopted. As far as we know, no studies have been carried out on semantic priming tasks in which ERPs have been used to investigate AoA effects. The present task was expected to evoke print-to-meaning mapping to retrieve the lexical-semantic encoding process, and thus would be a suitable task to examine the possible mapping from conceptual knowledge onto linguistic units, which was expected in the Arbitrary Mapping Hypothesis.

As reviewed above, previous accounts for the AoA effect have attributed it to the organization of the semantic system or to the way lemmas map onto the conceptual representation (e.g., Belke et al., 2005; Chen et al., 2007; Cuetos et al., 2009). Accordingly, one key ERP component relevant in the present study should be the N400, which indexes a process of semantic composition (Coch et al., 2013). Specifically, semantic unrelatedness or incongruity elicits a negative-going ERP which peaks around 400 ms, known as the N400, following the onset of the anomalous word, and the N400 amplitude is largest over central–parietal electrode sites (Kutas and Hillyard, 1984). In priming tasks, the repeated access to the same-shared representation usually elicit the reduction in the N400. The attenuated N400s observed are interpreted as a possible contribution of semantics (Feldman et al., 2009; Kielar and Joanisse, 2011). The other ERP component would be N100, reflecting attention allocation, working memory operation (Zhang et al., 2013), early perceptual processes such as feature integration and encoding, feature-mapping processes of speech sounds, the automatic lexical classification of a word and is relatively independent of the task (Spironelli and Angrilli, 2009) and a transitional processing stage between auditory and abstract phonological representations. So it is termed as “recognition potential.” The shorter latency of N100 usually represents higher mental ability. N100 latency and smaller N100 amplitudes have been interpreted as deficits in word recognition (Papageorgiou et al., 2009).

It was hypothesized that early acquired words should be more accessible than late-acquired words in the semantic judgment task. Therefore, early acquired words should cause larger N400 amplitudes. And regularity of the orthography-phonology (O-P) should modulate the AoA effect. When the orthography-phonology (O-P) is highly predictable, the AoA effect would be reduced. In contrast, when the O-P mapping is unpredictable, the AoA effect would be comparably much larger.

## Materials and Methods

### Participants

The participants were 28 students (25 female; 3 male) from a university in China. They were Chinese natives learning English as a foreign language. The participants’ mean age ranged from 18 to 20 (*M* = 18.71, *SD* = 0.85). They studied English for an average of 10.36 years (*SD* = 3.11). The mean AoA of L2 English was 8.36 (*SD* = 2.70). According to a 10-point self-rating scale (1 = not proficient and 10 = highly proficient), their mean English proficiency was 6.36 (*SD* = 0.95), and *M* = 7.70, *SD* = 1.21 for Chinese. Their language proficiency was indexed by the Gates Macginitie Reading Comprehension (Form 4, Level F), which had fourteen passages and 48 questions in total with a full score of 48. The participants were asked to finish this part within 30 min (Cronbach alpha = 0.87). The results showed the participants had an average of 25.71, *SD* = 6.99, maximum = 39, minimum = 13. All participants had normal or corrected to normal vision. It is confirmed that all studies conform to the relevant regulatory standards. The present study protocol was approved by the Experiment Ethics Committee by School of English, Beijing International Studies University. The participants gave their informed consent to participate in this study. They were compensated by money for their participation.

### Materials

Early acquired words, intermediate acquired words and late acquired words were, respectively, selected, respectively, from the 3rd-year primary school, the 2nd-year junior school, and the 2nd-year senior school English textbooks. The English textbooks were published by People’s Education Press affiliated by Ministry of Education in China. They were developed under the national guidelines on English education. It was expected the sampling would be representative of three major stages of AoA in terms of word onset acquisition time. Thus, the potential AoA effect was supposed to be generalized to the overall English learning situation in China. The stimuli comprised 90 English words, with 30 early AoA words (15 regular and 15 irregular words), 30 intermediate AoA words (15 regular and 15 irregular words), and 30 late AoA words (15 regular and 15 irregular words) (see **Appendix**). Regularity was defined on whether the pronunciation has a predictable orthography-to-phonology (O-P) correspondence. A written English word was regular if the pronunciation followed the O-P correspondence rules; and if the pronunciation did not confirm to the rules, it was an irregular word (Venezky, 1970). Two sets of semantic relatedness word pairs (related vs. unrelated) were generated for the 90 words (**Table 1**). For instance, for the semantic-related vs. unrelated word pairs, i.e., *pencil* – *pen* vs. *cup* – *pen*, the target “*pen”* would be the key word locked by ERP segments for data analysis. Another 180 English word pairs were generated as fillers with half of them being semantically related and the other half being unrelated in order to make the experimental material more variable and divert the participants’ attention from the targets. These fillers were not included in the data analysis since they were not controlled by variables like AoA, word length, frequency, etc.

**Table 1:** Sample of the experiment materials.

AoA	Regularity	Targets	Prime 1	Prime 2
			*Semantic related*	*Semantic unrelated*
Early	Regular	Pen	Pencil	Cup
	Irregular	Eye	Ear	Light
Intermediate	Regular	Lake	River	Sock
	Irregular	Read	Write	Cheat
Late	Regular	Dust	Ash	Closet
	Irregular	Source	Origin	Feature

Regular vs. irregular words across the three AoA groups were matched on word frequency, *F*(1,88) = 1.04, *p* = 0.31, number of letters, *F*(1,88) = 2.38, *p* = 0.13, and number of phonemes, *F*(1,88) = 0.13, *p* = 0.72. The word frequency was based on the *Corpus of Contemporary American English* (COCA^1^). Since globalization enables Chinese natives have access to American English, COCA could be deemed as a proper tool to present word frequency data. Besides, the best of our knowledge there was no suitable English word frequency corpus for the English textbooks in China. ANOVA analysis also found the three AoA groups were matched on frequency, *F*(2,87) = 1.28, *p* = 0.28. Two-tailed *t*-tests indicated that frequency was controlled between regular and irregular words across AoA groups. For early AoA, regular vs. irregular, *t*(14) = 0.93, *p* = 0.36; for intermediate AoA, regular vs. irregular, *t*(14) = 0.39, *p* = 0.69; for late AoA, regular vs. irregular, *t*(14) = -0.98, *p* = 0.34.

Word length across the three AoA groups was between 3 and 7 letters. Despite thorough perusal of the textbooks, word length was different across AoA groups [for early AoA, *M* = 3.93, *SD* = 0.78; for intermediate AoA, *M* = 4.43, *SD* = 0.68; for late AoA, *M* = 5.63, *SD* = 1.03; *F*(2,87) = 32.03, *p <* 0.001]. *Post hoc* (Turkey HSD) revealed that early AoA words were matched with intermediate AOA words on word length, *p* = 0.062. But late AoA words groups differed from early and intermediate AoA words in word length, *p*s *<* 0.001. Since it is well documented that word length has an effect on the N400 component (Osterhout et al., 2002), later interpretation of AoA effects would focus on the differences between the early and intermediate AoA words.

To control spillover effects from the prime words to the experimental words, which may contaminate the ERPs to the target words, priming words across AoA groups were controlled on word frequency, *F*(2,177) = 2.92, *p* = 0.06. Additionally, 15 participants who did not attend the ERP experiment evaluated offline the level of semantic-relatedness across the three AoA groups by 5-point Likert scale. The results showed the level of semantic relatedness was controlled across AoA groups for both semantic related and unrelated word pairs [For semantic related word pairs, *M*_early AoA_ = 4.04, *SD* = 0.43; *M*_intermediate_ _AoA_ = 3.82, *SD* = 0.62; *M*_late AoA_ = 3.84, *SD* = 0.56; *F*(2,87) = 1.40, *p* = 0.25. For semantic unrelated word pairs, *M*_early AoA_ = 1.81, *SD* = 0.62; *M*_intermediate AoA_ = 1.57, *SD* = 0.51; *M*_late AoA_ = 1.66, *SD* = 0.53; *F*(2,87) = 1.83, *p* = 0.17].

Additionally, the ratings confirmed the significant difference in the level of semantic relatedness between semantic related vs. unrelated word pairs (**Table 2**). Specifically, semantic related word pairs were higher in the level of semantic relatedness than semantic unrelated word pairs for the total word samples [*M*_semantic related_ = 3.90, *SD* = 0.55; *M*_semantic unrelated_ = 1.66, *SD* = 0.53; *F*(1,179) = 777.96, *p <* 0.001] and across the three AoA groups [For early AoA, *F*(1,59) = 260.61, *p <* 0.001; For intermediate AoA, *F*(1,59) = 238.57, *p <* 0.001; For late AoA, *F*(1,59) = 296.17, *p <* 0.001]. ANOVA analysis with AoA (early vs. intermediate vs. late) and semantic relatedness (related vs. unrelated) as two within-subject factors showed a main effect of semantic relatedness on semantic relation, *F*(2,58) = 895.22, *p <* 0.001. But there was neither AoA effect [*F*(2,58) = 2.67, *p* = 0. 07] nor interaction between AoA and semantic relatedness [*F*(1,29) = 0.01, *p* = 0.92] on the level of semantic relatedness.

**Table 2:** The ANOVA results of matched factors for targets and primes.

Factors	Early *M* (*SD*)	Intermediate *M* (*SD*)	Late *M* (*SD*)	*F*	*p*
Targets frequency	451677.13 (2039382.20)	49967.33 (125701.43)	11992.13 (10197.55)	1.28	0.28
Targets length	3.93 (0.78)	4.43 (0.68)	5.63 (1.03)	32.03	0.001
Semantic-related	4.04 (0.43)	3.82 (0.62)	3.84 (0.56)	1.4	0.25
Semantic-unrelated	1.81 (0.62)	1.57 (0.51)	1.66 (0.53)	1.83	0.17
Primes frequency	110437.28 (359272.10)	48310.97 (64377.83)	19232.80 (22432.20)	2.92	0.06
Sources					
Semantic relatedness				895.22	0.001
AoA				2.67	0.07
Semantic relatedness ^∗^ AoA				0.01	0.92

(1) For word length, Early AoA words = Intermediate AoA words, *p* = 0.062; (2) Semantic relatedness difference between semantic vs. unrelated word pairs found for the total sample [*F*(1,179) = 777.96, *p <* 0.001], and across AoA groups [For early AoA, *F*(1,59) = 260.61, *p <* 0.001; For intermediate AoA, *F*(1,59) = 238.57, *p <* 0.001; For late AoA, *F*(1,59) = 296.17, *p <* 0.001].

### Procedure

Participants viewed 180 word pairs (**Table 1** in Material Section) and 180 pairs of fillers. The 360 word pairs were presented in a pseudo-randomized order on the screen via the E-Prime software. There were two versions of the materials and the ordering of experimental word pairs and fillers was pseudorandomized. The word pairs were run in two separate sections. For each section, there were two blocks. During the process, the participants were seated one meter away from the computer screen. The room was sound-proof to establish a quiet environment so that the participants could concentrate. The experiment lasted for nearly 1 h with 3 breaks of 5–10 min each.

**FIGURE 1:**
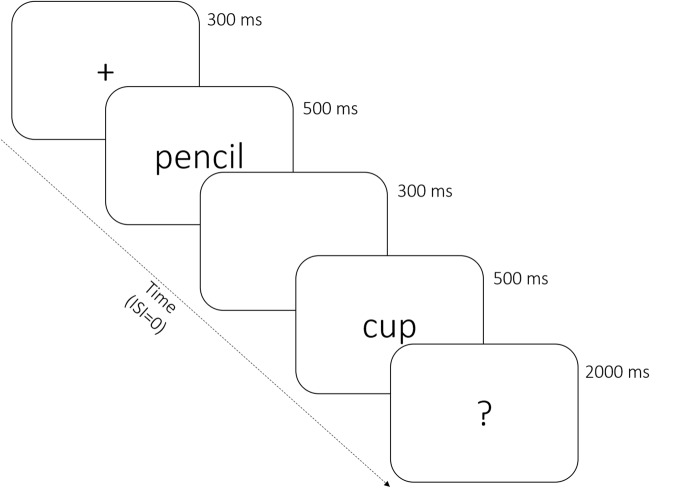
**Experimental sequence**.

Participants were required to decide whether words presented in pairs were related in meaning or not. After a prestimulus interval of 300 ms, the first word (prime) was flashed for 500 ms at fixation “+” followed by the second word (target) after an interstimulus interval of 300 ms. The duration of the second word presentation was also 500 ms. After the offset of the second word, a blank screen appeared for 300 ms, followed by a question mark “?” that served as a prompt for 2000 ms. When the prompt appeared, participants were supposed to respond by pressing either “1” or “2” (**Figure 1**). Participants gave their responses by pressing one of two buttons with their left or right index finger. Right and left hand response types were counterbalanced. Each participant first completed 10 practice trials, consisting of five semantic related and five semantic unrelated word pairs. All practice stimuli were similar to the experimental items.

### Data Collection

Continuous EEG was recorded from 64 active electrodes (Act-iCap, Brain Products GmbH, Munich) at standard international 10–20 system, referenced to bilateral mastoids and grounded to forehead. To control for vertical eye movements, a vertical electro-oculogram (VEOG) was recorded from Ag/AgCI electrodes placed closely above and below the left eye. Horizon eye movements were measured by a horizon electro-oculogram (HEGO) recorded from Ag/AgCI electrodes that placed at the outer canthus of each eye. All impedances were kept below 20 Ω during the experiment. EEG signals were bandpass filtered between 0.016 and 100 Hz, and amplified and digitized at a rate of 500 Hz using a BrainAmp amplifier (Brain Products GmbH, Munich). All EEG data were collected using Brain Vision Recorder (Brain Products GmbH, Munich).

### Data Analysis

The EEG data were processed offline using Brain Vision Analyzer 2. They were re-referenced to the mean of the left and right mastoids, and filtered with a 0.1 Hz high-pass filter to remove drifts and a 30 Hz filter to eliminate line noise. The artifacts caused by eye blinks, eye movements and muscular activity were removed using independent component analysis (ICA) (Onton and Makeig, 2006). ICA can blindly decompose multichannel EEG data into independent components (ICs) reflecting brain-generated EEG activities or irrelevant artifacts. Each IC has its activity time course and a set of projections to the recording electrodes as its scalp map. In this study, the artifact ICs were removed on the basis of visual inspection. ERPs were computed for 800 msec after the onset of the target word relative to a 200-msec prestimulus baseline.

Event-related potential waveforms were measured within N400 time windows determined by visual inspection of individual and group averages. Peak detection was performed automatically, time-locked to the latency of the peak at the electrode of maximal amplitude on the grand-average ERP. N100 and N400 were assessed by measuring the mean peak amplitude (average of non-rejected epochs from 0 to 800 ms after the onset of the target, calculated relative to a baseline from -200 to 0 ms) of ERPs for each participant.

Repeated measures ANOVAs were performed with within-subject factors of two levels of condition (C: correct, violation), two levels of hemisphere (H: left, right), three levels of word AoA (early, intermediate and late), and two levels of regularity (regular, irregular), two levels of semantic relatedness (related, unrelated) and three levels of anterior-posterior (frontal, central, and parietal). The three levels of anterior-posterior lobes involved the following sites: the frontal sites (in both left/right hemispheres: F1/F2, F3/F4, F5/F6, F7/F8), central sites (in both left/right hemispheres: C1/C2, C3/C4, C5/C6, C7/C8) and parietal sites (in both left/right hemispheres: P1/P2, P3/P4, P5/P6, P7/P8).

Following omnibus ANOVAs, additional analyses were performed in step-down fashion to isolate any significant interactions, collapsing across factors with which an interaction was not found. Reliable main effects and interactions were followed by simple effect analysis when appropriate. Main effects and interactions that involved AoA were reported, since AoA was of most theoretical interest in the present study. For all analyses, original degrees of freedom were reported. A Greenhouse-Geisser correction for sphericity was applied to *p* values when more than two levels of a factor were present (Greenhouse and Geisser, 1959). Any main effects not reported below were all non-significant (all *p*s > 0.05).

## Results

### Behavioral Results

**FIGURE 2:**
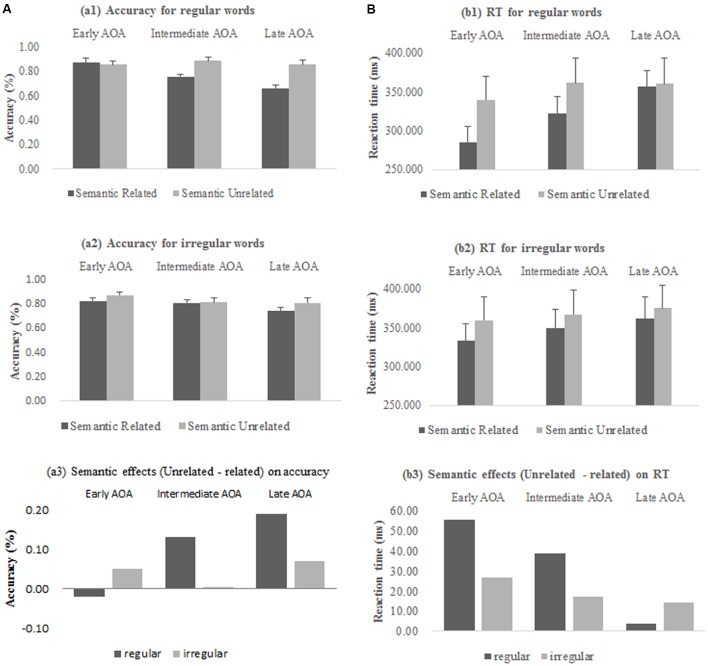
**Semantic effects on (A)** accuracy rates and **(B)** reaction times across different AoA.

After excluding three participants whose accuracy rate was lower than the below chance level (50%) and another one participant due to equipment failure during ERP recording, there were 24 effective participants. The participants had a relatively high accuracy rate when making semantic judgments on earlier learned words. The mean accuracy rate for early, intermediate, and late AoA were 0.86 (*SD* = 0.10), 0.82 (*SD* = 0.09), and 0.77 (*SD* = 0.11), respectively. Repeated measures ANOVAs were performed with AoA (early, intermediate, late), semantic relatedness (related vs. unrelated), and regularity (regular vs. irregular) as within-subject factors. The results revealed that there was a main effect of AoA [*F*(2,46) = 25.52, *p <* 0.001] and a marginal main effect of semantic relatedness [*F*(1,23) = 3.82, *p* = 0.06], an interaction between AoA and semantic relatedness [*F*(2,46) = 7.45, *p* = 0.003], and an interaction between semantic and regularity [*F*(1,23) = 10.08, *p* = 0.004] (**Figure 2A**). Simple effects analysis found AOA effects only in word related word pairs [*F*(2,46) = 20.16, *p <* 0.001]. There was a three-way interaction between AoA, semantic related and regularity [*F*(2,46) = 15.44, *p <* 0.001]. Simple effects analysis found AOA effects in semantic related words pairs for regular words [*M*_early AoA_ = 0.88, *SD* = 0.15, *M*_intermediate AoA_ = 0.76, *SD* = 0.09, *M*_late AoA_ = 0.66, *SD* = 0.14; *F*(2,46) = 28.78, *p <* 0.001] and for irregular words [*M*_early AoA_ = 0.83, *SD* = 0.14, *M*_intermediate AoA_ = 0.81, *SD* = 0.11, *M*_late AoA_ = 0.75, *SD* = 0.15; *F*(2,46) = 5.03, *p* = 0.01]. However, for semantic unrelated word pairs, AoA effects were found for irregular words [*M*_early AoA_ = 0.88, *SD* = 0.13, *M*_intermediate AoA_ = 0.82, *SD* = 0.19, *M*_late AoA_ = 0.81, *SD* = 0.20; *F*(2,46) = 6.50, *p* = 0.004], but not for regular words [*F*(2,46) = 1.32, *p* = 0.28].

Repeated measures ANOVAs on the mean RTs were performed with AoA (early, intermediate, late), semantic relatedness (related vs. unrelated), and regularity (regular vs. irregular) as within-subject factors. The results revealed that there was a main effect of AoA [*M*_earlyAoA_ = 329.26, *SD* = 121.83; *M*_intermediate AoA_ = 350.14, *SD* = 126.52; *M*_late AoA_ = 363.33, *SD* = 137.06; *F*(2,46) = 6.81, *p* = 0.007], a main effect of semantic relatedness [*M*_related_ = 334.60, *SD* = 23.02; *M*_unrelated_ = 360.56, *SD* = 29.06; *F*(1,23) = 6.41, *p* = 0.02] and a main effect of regularity [*M*_regular_ = 337.58, *SD* = 26.44; *M*_irregular_ = 357.58, *SD* = 25.66; *F*(1,23) = 5.54, *p* = 0.03] (**Figure 2B**). There was no other effect. Further analysis on the main AoA effect revealed that there was significant difference on reaction times between early and intermediate [*t*(23) = -2.95, *p* = 0.007], and between early and late AoA words [*t*(23) = -2.92, *p* = 0.008], but not between intermediate and late AoA words [*t*(23) = -1.54, *p* = 0.14]. **Figure 2** (a3 and b3) shows the semantic effects (measured by semantic unrelated condition minus semantic related condition) on accuracy and RTs across different AoA words.

### ERPs Results

**FIGURE 3:**
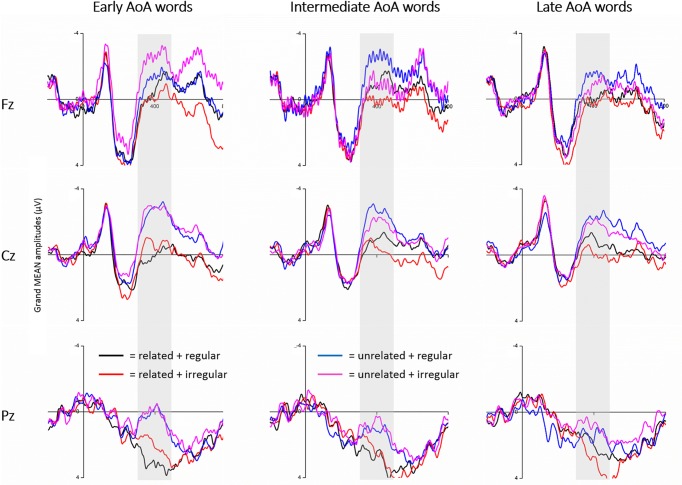
**Mean amplitudes in Fz, Cz, and Pz during processing words of different AoAs.** The 350–450 ms post stimulus is signaled by the gray square.

Grand-averaged ERPs time-locked to the onset of the target were exemplified at electrode sites Fz, Cz, Pz across AoA groups (**Figure 3**), and the waves represent the mean amplitudes evoked. Visual inspection revealed there were negative-going waves in the N100 (100–200 ms) and N400 (350–450 ms post-stimulus) time windows. Semantic unrelated word pairs evoked more negative going waves than the semantic related word pairs. Interestingly, N400 amplitudes were more negative going for early acquired words than those for late acquired words. Statistical analyses were performed on the mean peak amplitudes in the N100 and N400 time windows. Mean peak voltage amplitudes for N100 were detected within the time window of 80-150 ms, and N400 within time window of 350–450 ms post-stimulus. The average N100 latency was 122.42, *SD* = 16.29 and N400 latency was 404.92, *SD* = 74.99.

### Analysis on N100 Amplitudes

In the analyses of the N100 time windows, repeated measures ANOVAs were performed with within-subject factors of AoA (early, intermediate, late), semantic relatedness (related vs. unrelated), regularity (regular vs. irregular), hemisphere (left vs. right) and anterior-posterior (frontal vs. central vs. parietal). The results showed no main effects on AoA [*F*(2,46) = 1.17, *p* = 0.32], or semantic relatedness [*F*(1,23) = 0.019, *p* = 0.89], or regularity [*F*(1,23) = 1.91, *p* = 0.18], or hemisphere [*F*(1,23) = 0.65, *p* = 0.43] but a main effect of lobe [*F*(2,46) = 65.48, *p* = 0.001].

There was a marginal three-way interaction between semantic relatedness, hemisphere and lobe [*F*(2,46) = 3.22, *p* = 0.055]. There was a five-way interaction between AoA, semantic relatedness, regularity, hemisphere and lobe [*F*(4,92) = 3.01, *p* = 0.04]. Simple simple effects analysis revealed AoA effects on irregular words in semantic unrelated condition at the left parietal lobe [*M*_early AoA_ = 2.85, *SD* = 2.22; *M*_intermediate AoA_ = 2.13, *SD* = 2.47; *M*_late AoA_ = 1.53, *SD* = 2.90; *F*(2,46) = 5.74, *p* = 0.006]. Multiple comparison showed irregular early AoA words in semantic unrelated condition activated larger N100 amplitudes at the parietal lobe compared with intermediate AoA words [*t*(23) = 1.97, *p* = 0.061] and compared with late AoA words [*t*(23) = 3.65, *p* = 0.001], but intermediate AoA words did not differ from late AoA words [*t*(23) = 1.37, *p* = 0.18].

### Analysis on N400 Amplitudes

In the analyses of the N400 time windows, similar repeated measures ANOVAs were performed. The results showed there was a main effect of AoA [*M*_early AoA_ = 0.25, *SD* = 0.21; *M*_intermediate AoA_ = 0.32, *SD* = 0.20; *M*_late AoA_ = 0.62, *SD* = 0.20; *F*(2,46) = 4.01, *p* = 0.03], suggesting the early AoA words induced more negative-going N400 than intermediate and late AoA words. Multiple comparisons showed N400 was not different between early and intermediate AoA [*F*(1,23) = 0.29, *p* = 0.60], but the N400 was significantly different between early vs. late AoA [*F*(1,23) = 8.39, *p* = 0.008] and between intermediate vs. late AoA [*F*(1,23) = 4.33, *p* = 0.049]. There was a main effect of hemisphere [*F*(1,23) = 7.25, *p* = 0.01] and a main effect of lobe [*F*(2,46) = 22.09, *p <* 0.001], suggesting brain lobes were involved in word processing in a different way. There was a main effect of semantic relatedness [*M*_semantic related_ = 0.93, *SD* = 0.21; *M*_semantic unrelated_ = -0.14, *SD* = 0.22; *F*(1,23) = 45.11, *p <* 0.001], meaning the semantic unrelated word pairs evoked more negative going waves than the semantic related word pairs. ANOVA results confirmed the visual inspection of AoA and semantic-relatedness priming effects.

**FIGURE 4:**
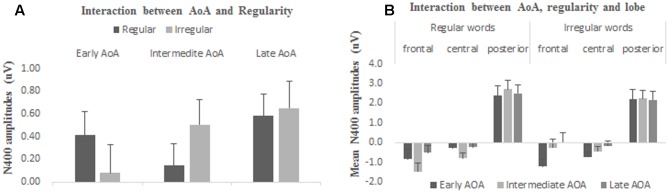
**Interaction (A)** between AoA and regularity and **(B)** between AoA, regularity and lobe.

There was a two-way interaction effect between regularity and lobe [*F*(2,46) = 5.53, *p* = 0.022] and between AoA and regularity [*F*(2,46) = 5.51, *p* = 0.009] (**Figure 4A**). Simple effect analysis found regularity effects in the intermediate and late AoA words, *p*s *<* 0.001, but not in the early AoA words, *p* = 0.75. And AoA effects were more pronounced in irregular words [*F*(2,46) = 5.61, *p* = 0.007] than regular words [*F*(2,46) = 3.41, *p* = 0.042].

There was a three-way interaction between regularity, hemisphere and anterior-posterior [*F*(2,46) = 3.57, *p* = 0.04] and a marginal three-way interaction between AoA, regularity and lobe [*F*(4,92) = 2.89, *p* = 0.06] (**Figure 4B**). Simple effect analysis found AoA effects for regular words in the frontal (*p* = 0.029) and central sites (*p* = 0.012), and for irregular words in the frontal (*p* = 0.007) and central sites (*p* = 0.025).

There was a four-way interaction between AoA, semantic relatedness, regularity and hemisphere, *F*(2,46) = 4.32, *p* = 0.022. Simple effect analysis showed AoA effects in the semantic unrelated condition at the left hemisphere for regular words [*F*(2,46) = 4.65, *p* = 0.01] and irregular words [*F*(2,46) = 3.11, *p* = 0.054], and at the right hemisphere for regular words [*F*(2,46) = 3.10, *p* = 0.055]. For the semantic related condition, AoA effects were found in the irregular word pairs at the right hemisphere [*F*(2,46) = 4.36, *p* = 0.02].

## General Discussion

The primary concern of the present study was to understand how Chinese native speakers processed early- vs. late-learned L2 English words. The target words in the experiment were selected, respectively, from required textbooks of primary, junior and senior high schools, generally representing the actual language input situation in the foreign language context in China. The AoA effect was found for this sample of Chinese-English bilinguals during L2 word processing. Accuracy for later acquired words processing was lower, and RT was longer (**Figure 2** a1–2/b1–2). These results were generally in line with previous findings (Brysbaert et al., 2000; Zevin and Seidenberg, 2002; Steyvers and Tenenbaum, 2005; Bowers and Kennison, 2011; Stolten et al., 2013). ERP data provided further evidence for the existence of the AoA effect, reflected in larger N100 and more negative N400 for early AoA words compared to later AoAs. Since N100 is “recognition potential,” larger N100 for early AoA words suggests more efficiency in word recognition (Papageorgiou et al., 2009). The results speak for advantages of learning a second language at an earlier age for the present sample of Chinese natives who learned English as a foreign language.

### AoA Effects more Pronounced for Semantic Related Conditions and for Irregular Word Pairs

One important finding was the interaction effect between AoA and semantic relatedness. In the present study, early AoA words have higher accuracy and shorter RT. The AoA effect was significant only for semantic related word pairs in terms of accuracy rates. Since the semantic-relatedness judgment involves encoding semantic representations and orthography-semantic mapping, the result suggests that the AoA effect involves semantic processing, and that the AoA determines the speed with which the semantic representations of concepts can be activated. The results seem to support the Semantic Locus Hypothesis that the concepts of late acquired words are constructed on the basis of the concept of early acquired words so that early acquired concepts are more accessible than late acquired concepts (Brysbaert et al., 2000; Belke et al., 2005). This theory explains the larger AoA effect for the picture-naming task (involving Semantic-Phonology mapping) than reading aloud the printed names of the same objects, and no AoA effect in a word-naming task (involved Orthography-Phonology mapping) (Belke et al., 2005). The results did not contradict the prediction by the Arbitrary Mapping hypothesis, which involves the mapping between orthographic and semantic representations. The AoA effect found only in the semantic related conditions suggests the AoA effect arises from the selection from the set of conceptually similar candidates as in the semantic-related word pairs. The main AoA effect on the mean N400 amplitudes further supports the argument that the locus of the AoA effect should derive from semantic representations. It is extensively accepted that the N400 is a negative-going potential between 300 and 500 ms that has been closely linked to the semantic processing (Kutas and Federmeier, 2001). Similarly, in Cuetos et al. (2009)’s study, participants read words of late and early AoA, and late AoA produced more negative amplitudes than early AoA at the late stage of about 400–610 ms window. The authors suggest that AoA influences processing at a semantic level or at the links between semantics and phonology.

### Different Patterns of Regularity Effects for the Three Groups of AoA Words

As to the second research question of how the O-P mapping rules are related with the AoA effect. The present results reveal different patterns of regularity effects for the three groups of AoA words. For early AoA words, irregular words were more difficult to process than regular words, as was reflected in the more negative-going N400 during processing irregular words; comparatively, irregular words were easier to process than regular words for intermediate and late AoA words (**Figure 4**). There was a tendency of a linear relationship between words AoAs and N400 for the irregular words. Specifically, irregular words of early AoAs activated more negative N400s than words of intermediate and late AoAs (**Figure 4A**). But the pattern was not applicable to regular words processing.

For one thing, the apparent AoA effect for irregular words suggests the AoA effect is more pronounced in words that involve arbitrary matching between orthography, phonology and semantic representations. This can be explained by the Arbitrary Mapping Hypothesis. According to the hypothesis, the AoA effect reflects the arbitrary nature of the mapping between input (e.g., orthography) and output (phonological or semantic) representations during the development of the lexical network. When the mapping between input and output is inconsistent, or arbitrary, AoA effects will be increased. In line with the Arbitrary Mapping Hypothesis, the mapping between the input and output in irregular words is unpredictable and arbitrary. This determines the size of the AoA effects for irregular words is pronounced than that in regular words. Irregular words could take less advantage of the shaped brain pattern than regular words. Thus, the present findings attributes the AoA effect to the arbitrary organization of the input-output mapping system.

For another thing, from the perspective of N400 amplitudes, the present study found a significant regularity effect for intermediate and late AoA words but not for early AoA words (see the ANOVA results). The regularity effect became slackened when AoA increased (**Figure 4**). The contrasting N400 patterns between regular and irregular words are likely to be rooted in the way of new word acquisition process. During the early period of word learning, the O-P mapping rules are on the way of establishing, and access to the regular words should be more energy consuming due to less efficiency in computing the O-P mapping rules. When the O-P rules are consolidated in the high school, access to the regular words should be easier, leading to less N400 effects during word processing. In this stage, for late AoA words processing, the direct mapping from orthographic inputs to their phonological representations did not appear to be a more efficient pathway for semantic judgment, as implicated in significantly longer reaction time. The O-P pathway with yet longer reaction time might suggest declined efficiency of the connections within the reading network for irregular/late acquired words. Rather than recourse to the O-P representations, the late AoA words tend to link the orthographic inputs directly to semantic processing. The shift in the selection of O-P mapping regularity rules might reflect neurocognitive changes during processing different AoA words. This process is similar to the findings in neuro-cognitive studies, which attribute the age effect to the changes in the plasticity of cognitive neural system (Ellis and Lambon Ralph, 2000). According to the cognitive plasticity account, the cognitive system has greater plasticity in learning early acquired-words, whereas learning later-acquired words is constrained by previous learning and should be modulated around existing representations (Belke et al., 2005; Chen et al., 2007; Hernandez and Li, 2007).

### Neural Representation for Different AoA Words

**FIGURE 5:**
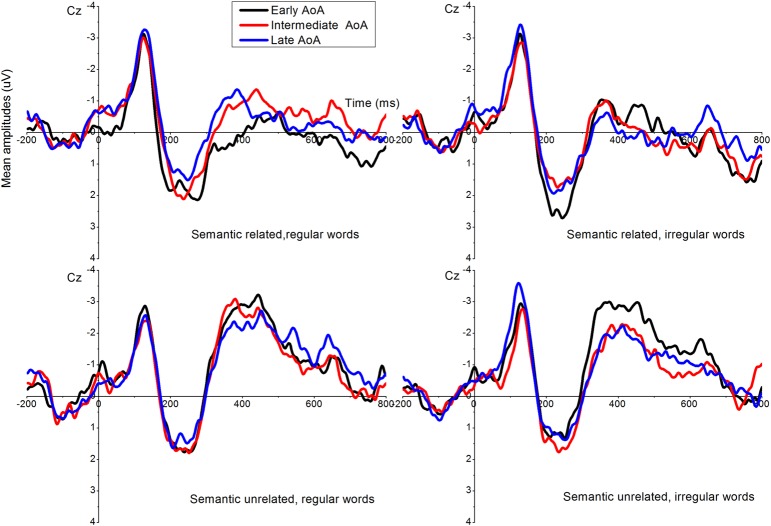
**Event-related potentials across AoA and conditions at Cz**.

A related finding in the present study was that the earlier AoA word processing activated more negative going N400s, which were accompanied with the faster RTs. The neural evidence further supports the Arbitrary Mapping Hypothesis (Ellis and Lambon Ralph, 2000) because the AoA effect was observed at the typical time window of N400 (i.e., 350–450 ms post-stimulus), indicating that AoA influenced processing at a semantic level (Brysbaert et al., 2000). The more negative going N400 for the words of earlier AoAs could be interpreted as more extended connections in between the semantic representations. This is also supported from the simulating organization structure of semantic networks in the mathematical model proposed by Steyvers and Tenenbaum (2005). According to that model, the order of meaning acquisition determines the connection strength of Nodes in the semantic network. The new concept Nodes in the semantic network would associate with the early acquired concept. The more connection means easier semantic access. The concept Node which had more connection was easy to be connected. Surprisingly, in the sematic related condition, a smallest N400 was elicited in the central and posterior only for early regular AoA words relative to intermediate and late AoA words (**Figure 5**). We tentatively argue that early acquired regular words in the semantic relatedness condition involve less semantic processing, or alternatively lexicons of early regular words have stronger connections with semantics representations. Accordingly, early acquired regular words benefit less from semantic priming in the semantic-related condition. Similar evidence also comes from less involvement of left IFG (or semantic processing) for regular syntactic processing compared with irregular syntactic processing (e.g., Desai et al., 2006; Hernandez et al., 2007).

Previous research has found activation in the precuneus area in response to early acquired words (Fiebach et al., 2003). Neural imaging studies (Hernandez and Fiebach, 2006; Cuetos et al., 2009) compared early AoA and a baseline, and found bilateral activation at the precuneus, as well as the inferior parietal lobe, the frontal lobe, the frontal inferior gyrus (in the posterior superior area) and the premotor cortex. Bilateral frontal medial lobe, the left precentral gyrus and the insula were activated during processing late acquired words. In the present study, AoA effects for regular and irregular words were found in the frontal and central sites. Therefore, the most relevant results, using different tasks, seem to indicate that the AoA effect is associated with the system of distributed knowledge (Hernandez and Fiebach, 2006).

In sum, during semantic processing, earlier acquired words are easier and quicker to process, suggesting an advantage of early learning on word processing. The AoA effect is more likely to appear in semantic related word pairs and in irregular words, suggesting the locus of the AoA effect to the arbitrary mapping between word forms and conceptual representations. The regularity of orthography-phonology mapping rules is differentially associated with words AoAs, which reflects different processing strategies in word acquisition. The differential processing strategies implicate neurocognitive changes in word acquisition in different stages of word acquisition. Last, neuronal evidence also supports the involvement of semantics in the AoA effect with early learned words having more semantic connections than the late acquired words.

## Conclusion

As far as we know, this is the first research conducted using the ERP paradigm to measure the AoA effect on L2 English word processing for Chinese natives who learn English as a foreign language in China. The present study found the age of word acquisition played an important role in L2 word acquisition for Chinese native learners of English. This was reflected in the difference in word processing accuracy, speed and neural representation between early, intermediate and late AoA words. Regularity of the orthography-phonology-semantic mapping contributed to the AoA effect during word processing. The findings suggest the AoA effect seems to arise from the arbitrary mapping between conceptual knowledge onto linguistic units (orthography and phonology) (Belke et al., 2005). Earlier acquired words have more semantic connections which facilitate word processing. The differential AoA effect for regular vs. irregular words might be subjected to different neurocognitive processing strategies.

The present study has some limitations nonetheless. First, the selection of early, intermediate vs. late acquired words followed a general demarcation based on authoritative mandated textbooks for the three stages of word learning. It still cannot provide a complete picture of words AoA for learners. Second, even though some variables such as word frequency, number of letters were controlled, some variables such as imageability and concreteness might have some influence on the results. For instance, the present study did not balance the language features such as concreteness across AoA groups. Even though earlier stage of language learning in reality is characterized by the concrete words, the concreteness of words could be a factor confounding the N400 effect. Future studies on the AoA effect should take into consideration the language features such as concreteness of words, parts of speech and so on, to see how these features modulate the AoA effect during word learning. Despite the limitations, the present study may shed light on the locus of the AoA effects the neuronal-cognitive mechanism underlying the AoA effects for second language learners. A strong implication of these findings is that for Chinese-English bilinguals, second language words processing might be relatively fast and successful when they are acquired at an early stage. The findings were supposed to reflect the AoA effect on behavioral and neuronal representation for the sample of Chinese natives who learn English as a foreign language.

## Author Contributions

JX designed the experiment and wrote the manuscript. FM-R drew the figures and revised the manuscript. TL and XP prepared the paper and collected the data.

## Conflict of Interest Statement

The authors declare that the research was conducted in the absence of any commercial or financial relationships that could be construed as a potential conflict of interest.

## Footnotes

1 http://corpus.byu.edu/coca/

## Appendix

### Early AoA words

Regular: pen, three, red, man, and, cat, cake, white, boy, long, peach, nice, green, nine, deer. Irregular: eye, pink, chick, apple, bear, head, too, water, four, real, ball, tea, mouth, ear, foot.

### Intermediate AoA words

Regular: bell, bench, bake, feed, lake, brave, rich, milk, river, kill, cost, voice, time, tree, boat. Irregular: fear, seat, autumn, laugh, read, clever, most, allow, alien, extra, area, angry, copy, meter, cow.

### Late AoA words

Regular: solid, shade, victory, paint, dust, insist, recite, fortune, candy, quote, predict, bite, employ, mark, chat. Irregular: harmony, editor, glimpse, elect, source, poem, wound, arrival, basis, require, false, ignore, supply, sample, advise.

## References

[B1] Belke E. Brysbaert M. Meyer A. S. Ghyselinck M. (2005). Age of acquisition effects in picture naming: evidence for a lexical-semantic competition hypothesis. *Cognition* 96 B45–B54. 10.1016/j.cognition.2004.11.006 15925568

[B2] Binder J. R. Desai R. H. (2011). The neurobiology of semantic memory. *Trends Cogn. Sci.* 15 527–536. 10.1016/j.tics.2011.10.001 22001867 PMC3350748

[B3] Birdsong D. (2005). “Interpreting age effects in second language acquisition,” in *Handbook of bilingualism:Psycholinguistic Approaches*, eds Kroll J. F. Groot A. M. B. de (New York, NY: Oxford University Press), 109–127.

[B4] Bowers J. M. Kennison S. M. (2011). The role of age of acquisition in bilingual word translation: evidence from spanish-english bilinguals. *J. Psycholinguist. Res.* 40 275–289. 10.1007/s10936-011-9169-z 21687967

[B5] Brown G. D. A. Watson F. L. (1987). First in, first out. Word learning age and spoken word frequency as predictors of word familiarity and word naming latency. *Mem. Cogn.* 15 208–216. 10.3758/BF03197718 3600260

[B6] Brysbaert M. Van Wijnendaele I. De Deyne S. (2000). Age-of-acquisition of words is a significant variable in semantic tasks. *Acta Psychol.* 104 215–226. 10.1016/S0001-6918(00)00021-4 10900706

[B7] Chen B. G. Zhou H. X. Dunlap S. Perfetti C. A. (2007). Age of acquisition effects in reading Chinese: evidence in favour of the arbitrary mapping hypothesis. *Br. J. Psychol.* 98(Pt 3), 499–516. 10.1348/000712606X165484 17705943

[B8] Coch D. Bares J. Landers A. (2013). ERPs and morphological processing: the N400 and semantic composition. *Cogn. Affect. Behav. Neurosci.* 13 355–370. 10.3758/s13415-012-0145-3 23271630

[B9] Coltheart M. Rastle K. Perry C. Langdon R. Ziegler J. (2001). The DRC model: a model of visual word recognition and reading aloud. *Psychol. Rev.* 108 204–258. 10.1037/0033-295X.108.1.204 11212628

[B10] Cuetos F. Barbon A. Urrutia M. Dominguez A. (2009). Determining the time course of lexical frequency and age of acquisition using ERP. *Clin. Neurophysiol.* 120 285–294. 10.1016/j.clinph.2008.11.003 19101202

[B11] D’Angiulli A. Griffiths G. Marmolejo-Ramos F. (2015). Neural correlates of visualizations of concrete and abstract words in preschool children: a developmental embodies approach. *Front. Psychol.* 6:856 10.3389/fpsyg.2015.00856 PMC4484221 26175697

[B12] DeKeyser R. M. (2013). Age effects in second language learning: stepping stones toward better understanding. *Lang. Learn.* 63 52–67. 10.1111/j.1467-9922.2012.00737.x

[B13] Desai R. Conant L. L. Waldron E. Binder J. R. (2006). FMRI of past tense processing: the effects of phonological complexity and task difficulty. *J. Cogn. Neurosci.* 18 278–297. 10.1162/jocn.2006.18.2.278 16494687 PMC1679797

[B14] Ellis A. W. Lambon Ralph M. A. (2000). Age of acquisition effects in adult lexical processing reflects loss of plasticity in maturing systems: insights from connectionist networks. *J. Exp. Psychol.* 26 1103–1123. 10.1037/0278-7393.26.5.1103 11009247

[B15] Feldman L. B. O’Connor P. A. del Prado Martín F. M. (2009). Early morphological processing is morphosemantic and not simply morph-orthographic: a violation of form-then-meaning accounts of word recognition. *Psychon. Bull. Rev.* 16 684–691. 10.3758/PBR.16.4.684 19648453 PMC2883124

[B16] Fiebach C. J. Friederici A. D. Müller K. von Cramon D. Y. Hernandez A. (2003). Distinct brain representations for early and late learned words. *Neuroimage* 19 1627–1637. 10.1016/S1053-8119(03)00227-1 12948717

[B17] Flege J. E. Yeni-Komshian G. H. Liu S. (1999). Age constraints on second-language acquisition. *J. Mem. Lang.* 41 78–104. 10.1006/jmla.1999.2638

[B18] Greenhouse S. W. Geisser S. (1959). On methods in the analysis of profile data. *Psychometrika* 24 95–112. 10.1007/BF02289823

[B19] Hernandez A. E. Fiebach C. J. (2006). The brain bases of reading late learned words: evidence from functional MRI. *Vis. Cognit.* 13 1027–1043. 10.1016/j.neuropsychologia.2008.01.020

[B20] Hernandez A. E. Hofmann J. Kotz S. A. (2007). Age of acquisition modulates neural activity for both regular and irregular syntactic functions. *Neuroimage* 36 912–923. 10.1016/j.neuroimage.2007.02.055 17490895 PMC1995424

[B21] Hernandez A. E. Li P. (2007). Age of acquisition: its neural and computational mechanisms. *Psychol. Bull.* 133 638–650. 10.1037/0033-2909.133.4.638 17592959

[B22] Huang B. H. (2014). The effects of age on second language grammar and speech production. *J. Psycholinguist. Res.* 43 397–420. 10.1007/s10936-013-9261-7 23975257

[B23] Iossifova R. Marmolejo-Ramos F. (2013). When the body is time: spatial and temporal deixis in children with visual impairments and sighted children. *Res. Dev. Disabil.* 34 2173–2184. 10.1016/j.ridd.2013.03.030 23643770

[B24] Kielar A. Joanisse M. F. (2011). The role of semantic and phonological factors in word recognition: an ERP cross-modal priming study of derivational morphology. *Neuropsychologia* 49 161–177. 10.1016/j.neuropsychologia.2010.11.027 21129390

[B25] Klein D. Moka K. Chen J.-K. Watkins K. E. (2014). Age of language learning shapes brain structure:A cortical thickness study of bilingual and monolingual individuals. *Brain Lang.* 131 20–24. 10.1016/j.bandl.2013.05.014 23819901

[B26] Ku Y. M. Anderson R. C. (2001). Chinese children’s incidental learning of word meanings. *Contemp. Educ. Psychol.* 26 249–266. 10.1006/ceps.2000.1060 11273659

[B27] Kutas M. Federmeier K. D. (2001). Electrophysiology reveals semantic memory use in language comprehension. *Trends Cogn. Sci.* 4 463–470. 10.1016/S1364-6613(00)01560-6 11115760

[B28] Kutas M. Hillyard S. A. (1984). Brain potentials during reading reflect word expectancy and semantic association. *Nature* 307 161–163. 10.1038/307161a0 6690995

[B29] Lee C.-Y. Tsai J.-L. Su E. C.-I. Tzeng O. J. L. Hung D. L. (2005). Consistency, regularity, and frequency effects in naming Chinese characters. *Lang. Linguist.* 6 75–107.

[B30] Li Y. Kang J. S. (eds) (1993). *Analysis of Phonetics of the Ideophonetic Characters in Modern Chinese.* Shanghai: Shanghai Education Publisher.

[B31] Llanes A. Muñoz C. (2013). Age effects in a study abroad context: children and adults studying abroad and at home. *Lang. Learn.* 63 1–28. 10.1111/j.1467-9922.2012.00731.x

[B32] Lu Z. Tu L. (2010). The L2 acquisition age and the proficient Chinese-English bilinguals mental lexicon representation: evidence from a bilingual stroop task. *J. Foreign Lang.* 33 47–56.

[B33] Marcotte K. Ansaldo A. I. (2014). Age-related behavioural and neurofunctional patterns of second language word learning: different ways of being successful. *Brain Lang.* 135 9–19. 10.1016/j.bandl.2014.04.004 24880754

[B34] Mayberry R. I. Lock E. (2003). Age constraints on first versus second language acquisition: evidence for linguistic plasticity and epigenesis. *Brain Lang.* 87 369–384. 10.1016/s0093-934x(03)00137-8 14642540

[B35] Montrul S. Foote R. (2014). Age of acquisition interactions in bilingual lexical access: a study of the weaker language of L2 learners and heritage speakers. *Int. J. Biling.* 18 274–303. 10.1177/1367006912443431

[B36] Muñoz C. (2008). Age-related differences in foreign language learning. Revisiting the empirical evidence. *Int. Rev. Appl. Linguist. Lang. Teach.* 46 197–220.

[B37] Onton J. Makeig S. (2006). Information-based modeling of event- related brain dynamics. *Prog. Brain Res.* 159 99–120. 10.1016/S0079-6123(06)59007-7 17071226

[B38] Osterhout L. Allen M. McLaughlin J. (2002). Words in the brain: lexical determinants of word-induced brain activity. *J. Neurolinguistics* 15 171–187. 10.1016/S0911-6044(01)00036-7

[B39] Papageorgiou C. Giannakakis G. A. Nikita K. S. Anagnostopoulos D. Papadimitriou G. N. Rabavilas A. (2009). Abnormal auditory ERP N100 in children with dyslexia: comparison with their control siblings. *Behav. Brain Funct.* 5 1–10. 10.1186/1744-9081-5-26 19558644 PMC2707365

[B40] Pfenninger S. E. Singleton D. (2016). Affect trumps age: a person-in-context relational view of age and motivation in SLA. *Second Lang. Res.* 32 311–345. 10.1177/0267658315624476

[B41] Rakhlin N. Hein S. Doyle N. Hart L. Macomber D. Ruchkin V. (2015). Language development of internationally adopted children: adverse early experiences outweigh the age of acquisition effect. *J. Commun. Disord.* 57 66–80. 10.1016/j.jcomdis.2015.08.003 26385197

[B42] Saito K. (2013). Age effects on late bilingualism: the production development of /ɺ/ by high-proficiency Japanese learners of English. *J. Mem. Lang.* 69 546–562. 10.1016/j.jml.2013.07.003

[B43] Spironelli C. Angrilli A. (2009). Developmental aspects of automatic word processing: language lateralization of early ERP components in children, young adults and middle-aged subjects. *Biol. Psychol.* 80 35–45. 10.1016/j.biopsycho.2008.01.012 18343558

[B44] Steyvers M. Tenenbaum J. B. (2005). The language scale structure of semantic networks: statistical analyses and a model of semantic growth. *Cogn. Sci. Soc.* 29 41–78. 10.1207/s15516709cog2901_3 21702767

[B45] Stolten K. Abrahamsson N. Hyltenstam K. (2013). Effects of age of learning on voice onset time: categorical perception of swedish stops by near-native L2 speakers. *Lang. Speech* 57 425–450. 10.1177/0023830913508760 25536842

[B46] Venezky R. L. (1970). *The Structure of English Orthography.* The Hague: Mouton. 10.1515/9783110804478

[B47] Wartenburger I. Heekeren H. R. Abutalebi J. Cappa S. F. Villringer A. Peran D. (2003). Early setting of grammatical processing in the bilingual brain. *Neuron* 37 159–170. 10.1016/S0896-6273(02)01150-9 12526781

[B48] Weber-Fox C. M. Neville H. J. (1996). Maturational constraints on functional specializations for language processing: ERP and behavioral evidence in bilingual speakers. *J. Cogn. Neurosci.* 8 231–256. 10.1162/jocn.1996.8.3.231 23968150

[B49] Weekes B. S. Chan A. Kwok J. S. W. Tan L. H. Jin Z. (2004). AoA effects on Chinese language processing: an fMRI study. *Brain Lang.* 91 33–34. 10.1016/j.bandl.2004.06.020

[B50] Wei M. Joshi A. A. Zhang M. Mei L. Manis F. R. He Q. (2015). How age of acquisition influences brain architecture in bilinguals. *J. Neurolinguist.* 36 35–55. 10.1016/j.jneuroling.2015.05.001 PMC5045052 27695193

[B51] Xue J. Marmolejo-Ramos F. Pei X. (2015). The linguistic context effects on the processing of body-object interaction words: an ERP study on second language learners. *Brain Res.* 1613 37–48. 10.1016/j.brainres.2015.03.050 25858488

[B52] Xue J. Yang J. Zhao Q. (2014). Chinese-English bilinguals processing temporal-spatial metaphor. *Cogn. Process.* 15 269–281. 10.1007/s10339-014-0621-5 24889328

[B53] Yang C. L. Perfetti C. A. Schmalhofer F. (2007). Event-related potential indicators of text integration across sentence boundaries. *J. Exp. Psychol. Learn. Mem. Cogn.* 33 55–89. 10.1037/0278-7393.33.1.55 17201554

[B54] You W. Chen B. Dunlap S. (2009). Frequency trajectory effects in Chinese character recognition: evidence for the arbitrary mapping hypothesis. *Cognition* 110 39–50. 10.1016/j.cognition.2008.08.004 19091311

[B55] Zevin J. D. Seidenberg M. S. (2002). Age of acquisition effects in word reading and other tasks. *J. Mem. Lang.* 47 1–29. 10.1006/jmla.2001.2834

[B56] Zhang E. Luo J. Zhang J. Wang Y. Zhong J. Li Q. (2013). Neural mechanisms of shifts of spatial attention induced by object words with spatial associations: an ERP study. *Exp. Brain Res.* 227 199–209. 10.1007/s00221-013-3500-x 23575954

